# Quantification of *Slackia* and *Eggerthella* spp. in Human Feces and Adhesion of Representatives Strains to Caco-2 Cells

**DOI:** 10.3389/fmicb.2016.00658

**Published:** 2016-05-09

**Authors:** Gyu-Sung Cho, Felix Ritzmann, Marie Eckstein, Melanie Huch, Karlis Briviba, Diana Behsnilian, Horst Neve, Charles M. A. P. Franz

**Affiliations:** ^1^Department of Microbiology and Biotechnology, Max Rubner-Institut, Federal Research Institute of Nutrition and FoodKiel, Germany; ^2^Department of Safety and Quality of Fruit and Vegetables, Max Rubner-Institut, Federal Research Institute of Nutrition and FoodKarlsruhe, Germany; ^3^Department of Physiology and Biochemistry of Nutrition, Max Rubner-Institut, Federal Research Institute of Nutrition and FoodKarlsruhe, Germany; ^4^Department of Food Technology and Bioprocess Engineering, Max Rubner-Institut, Federal Research Institute of Nutrition and FoodKarlsruhe, Germany

**Keywords:** coriobacteria, microbiota, gastrointestinal, adherence, cell culture

## Abstract

*Eggerthella* and *Slackia* spp. are gut associated bacteria that have been suggested to play roles in host lipid and xenobiotic metabolism. A quantitative PCR method for the selective enumeration of bacteria belonging to either the genus *Eggerthella* or *Slackia* was developed in order to establish the numbers of these bacteria occurring in human feces. The primers developed for selective amplification of these genera were tested first in conventional PCR to test for their specificity. Representative species of *Eggerthella* and *Slackia*, as well as closely related genera of the *Coriobacteriia*, were included in the investigation. The selected primers were shown to be capable of specific amplification of species of the genera *Eggerthella* and *Slackia*, but not all species of the genera may be amplified by the respective primers. Their use in qPCR experiments to assess the levels of *Slackia equolifaciens* and *Eggerthella lenta* in the feces of 19 human volunteers showed they occurred at mean counts of 7 × 10^5^ and 3.1 × 10^5^ CFU/g for *Eggerthella* spp. and *Slackia* spp., respectively. Electron microscopy investigations showed that while *E. lenta* cells exhibited slender and very regular shaped rods, *Slackia* cells showed a remarkably pleomorphic phenotype. Both species did not appear to have fimbriae or pili. Some *S. equolifaciens* cells showed a characteristic “ribbon” of presumably extracellular material around the cells, particularly at the areas of cell division. The two species also differed markedly in their adhesion behavior to Caco-2 cells in cell culture, as *E. lenta* DSMZ 15644 showed a high adhesion capacity of 74.2% adherence of the bacterial cells added to Caco-2 cells, while *S. equolifaciens* DSM 24851^T^ on the other hand showed only low adhesion capability, as 6.1% of bacterial cells remained bound. Speculatively, this may imply that the ecological compartments where these bacteria reside in the gut may be different, i.e., *E. lenta* may be associated more with the gut wall, while *Slackia* may be free living in the lumen.

## Introduction

The *Coriobacteriia* are a diverse group of Gram-positive, non-sporeforming and non-motile bacteria and species belonging to this group of bacteria commonly occur in the human gut. The class *Coriobacteriia* is one of the deepest branching lineages within the phylum *Actinobacteria*, branching in the proximity of the phylum *Firmicutes* (König, 2012; Gupta et al., 2013). Recently, Gupta et al. (2013) proposed a division of the species of the *Coriobacteriia* into two orders, viz. the C*oriobacteriales* and *Eggerthellales*, containing the three families *Coriobacteriaceae*, *Atopobiaceae*, and *Eggerthellaceae*.

Coriobacteria are common members of the human and animal gut microbiota (Eckburg et al., 2005; Arumugam et al., 2011) and amongst these, *Collinsella* and *Eggerthella* are among the top five most abundant genera of the phylum *Actinobacteria* in the human gut. Harmsen et al. (2000) developed 16S rRNA-targeted probes for the coriobacteria and showed that these bacteria are present in human feces in numbers of about 1 × 10^10^ per gram feces. However, the probe used targeted what the authors termed the ‘*Atopobium* cluster’ which includes the genera *Collinsella, Atopobium, Coriobacterium*, and *Cryptobacterium.* Using this probe, the authors found ‘*Atopobium*-cluster’ counts of between 8.6 × 10^9^ up to 1.1 × 10^10^ in human feces (per gram dry weight). Thorasin et al. (2015) further investigated the coverage of the probe and showed that in addition to these, the genera *Olsenella, Paraeggerthella, Gordonibacter*, and ‘*Enorma*’ were also targeted by the probe.

A probe which targeted both the *Collinsella* and *Coriobacterium* species (‘*Collinsella*-group’ probe) showed lower counts of between 4.1 × 10^9^ to 7.7 × 10^9^ bacteria per gram dry weight feces.

Investigations have shown gut-associated coriobacteria to have a role in gut health. For example, the *Coriobacteriaceae* have been repeatedly linked to positive effects in host lipid metabolism, and Claus et al. (2011) showed an association between *Coriobacteriaceae*, particularly bacteria related to *Eggerthella lenta* and *Paraeggerthella hongkongensis* on triglyceride levels in mice and concluded that these bacteria are involved in the stimulation of a major hepatic detoxification activity. Strong positive links were also determined with plasma non-high-density-lipoprotein (non-HDL) in hamsters (Martínez et al., 2009). Martínez et al. (2013) showed that the connection between *Coriobacteriaceae* and *Erysipelothrichaceae* and host lipid metabolism could be caused by a metabolic phenotype, i.e., cholesterol excretion, which affects the microbiota. On the other hand, a study which investigated the gut colonization of axenic mice with a typical gut microbiota, and which compared microbiota colonization patterns with hepatic metabolomics profiles, could show that the *Coriobacteriaceae* are strongly associated with the metabolism of xenobiotics (Claus et al., 2011). Furthermore, *Collinsella aerofaciens* has been shown to be a member of the core microbiome (Qin et al., 2010).

*Eggerthella lenta*, *Asaccharobacter celatus*, *Adlercreutzia equolifaciens*, and *Slackia equolifaciens* belong to the *Eggerthellaceae* and strains of these species have been shown to play a role in the transformation of bioactive secondary plant compounds such as daidzein from soy, or *trans*-resveratrol from grape vine (Matthies et al., 2009; Tsuji et al., 2012; Bode et al., 2013; Schröder et al., 2013). For example, daidzein was shown to be transformed into dihydrodaidzein and equol by *S. isoflavoni*-covertans HE8 (Matthies et al., 2009) and the genes involved were cloned and sequenced (Schröder et al., 2013). Equol has a higher binding affinity to human estrogen receptors α and β than daidzein (10–80 times) and induces transcription more strongly than any other isoflavone (Decroos et al., 2005). Bode et al. (2013) showed that *E. lenta*, *Adlercreutzia equolifaciens*, and *S. equolifaciens* strains produced the metabolite dihydroresveratrol from *trans*-resveratrol *in vitro*. The biological activity of this metabolite, and hence the importance of this biotransformation has not yet been well studied. *Eggerthella lenta* was shown to metabolize the cardiac drug digoxin (Haiser et al., 2013), indicating that members of the gut microbiota may play a role in inactivation of pharmaceuticals in the gut.

Although the numbers of actinobacteria or coriobacteria in the human intestine have been determined before by using oligonucleotide probes (Harmsen et al., 2000), there is currently not much information on specifically the numbers of bacteria belonging to the genera *Slackia* and *Eggerthella* spp. in the human intestine, despite their possible importance with respect to production of bioactives in the gut. The aim of this study therefore, was to develop a genus-specific, quantitative PCR method for the specific enumeration of these bacterial genera in human feces. Furthermore, as not much is known about the ecology of these bacteria, the study also aimed to assess whether these bacteria possibly adhere to gut epithelial cells. Lastly, we used scanning and transmission electron microscopy to characterize the cell morphologies.

## Materials and Methods

### Bacterial Strains and Growth Conditions

The bacteria and growth media used in this study are shown in **Table 1**. Apart from coriobacteria belonging to the genera *Adlercreutzia, Atopobium, Collinsella, Eggerthella, Enterorhabdus, Gordonibacter*, and *Slackia*, other bacteria used as negative controls for qPCR experiments included *Escherichia coli, Lactobacillus plantarum*, strains BFE 5092 and 299v*, Bacteroides uniformis* and *Ruminococcus obeum* strains (**Table 1**). All bacterial strains were grown at 37°C. With the exception of *E. coli* DH5α and *L. plantarum* strains, all bacteria were grown anaerobically, either in Hungate tubes under nitrogen (coriobacteria) or in a Don Whitley A45 anaerobic chamber (Shipley, West Yorkshire, UK; *Bacteroides uniformis* DSM 6597, *Ruminococcus obeum* DSM 25238) under 80% N_2_, 10% CO_2_, and 10% H_2_.

**Table 1:** Bacteria belonging to the coriobacteria-group, as well as other control strains used in this study.

Strain	Medium	Growth condition
*Adlercreutzia equolifaciens* DSM 19450^T^	339^2^	Anaerobic condition^1^, 37°C
*Atopobium parvulum* DSM 20469^T^	104^3^	Anaerobic condition^1^, 37°C
*Collinsella intestinalis* DSM 13280^T^	104^3^	Anaerobic condition^1^, 37°C
*Eggerthella lenta* DSM 15644	339^2^	Anaerobic condition^1^, 37°C
*Eggerthella lenta* DSM 2243^T^	339^2^	Anaerobic condition^1^, 37°C
*Enterorhabdus mucosicola* DSM 19490^T^	339^2^	Anaerobic condition^1^, 37°C
*Faecalibacterium prausnitzii* DSM 17677	339^2^	Anaerobic condition^1^, 37°C
*Gordonibacter pamelaeae* DSM 19378^T^	339^2^	Anaerobic condition^1^, 37°C
*Slackia equolifaciens* DSM 24851^T^	339^2^	Anaerobic condition^1^, 37°C
*Escherichia coli* DH5alpha	LB^5^	Aerobic, 37°C, 180 rpm^4^
*Lactobacillus plantarum* BFE 5092	MRS^5^	Aerobic, 30°C
*Lactobacillus plantarum* 299v	MRS	Aerobic, 30°C
*Bacteroides uniformis* DSM 6597^T^	104^3^	Anaerobic condition^1^, 37°C
*Ruminococcus obeum* DSM 25238^T^	104^3^	Anaerobic condition^1^, 37°C

*^1^(80% N_2_, 10% CO_2_, and 10% H_2_) ^2^Deutsche Sammlung von Mikroorganismen (DSM) medium 339, also known as Wilkins–Chalgren anaerobe broth. ^3^DSM medium 104, also known as modified PYG medium. ^4^rpm, revolutions per minute on a shaker. ^5^LB, Luria–Bertani (Carl Roth, Karlsruhe, Germany); MRS, de Man, Rogosa and Sharpe (Merck, Darmstadt, Germany).*

### Scanning and Transmission Electron Microscopy

Stationary-phase liquid cultures of *E. lenta* DSMZ 15644 and *S. equolifaciens* DSMZ 24851^T^ grown as described above were used for electron microscopy. Samples were prepared for scanning electron microscopy as described before (Vinderola et al., 2012) and viewed in a XL30 scanning electron microscope (FEI Company, Eindhoven) in high vacuum mode with a secondary electron detector at an acceleration voltage of 15 kV. Furthermore, *S. equolifaciens* DSMZ 24851^T^ suspensions (10^7^ cells/mL) were deposited onto silicon substrates and fixed with 2.5% (v/v) glutaraldehyde in 0.1 M sodium phosphate buffer pH 7.4 under anaerobic conditions. The samples were then post-fixed with 0.1% (w/v) OsO_4_, subjected to an ethanol gradient (25–100%) and finally critical point dried (BAL-TEC CPD 030, Balzers, Liechtenstein). The silicon substrates with the fixed and dried sample were mounted onto aluminum stubs with double coated carbon conductive tabs. Samples were viewed using a FEG-SEM Quanta 250 (FEI, Czech Republic) equipped with an Everhart–Thornley detector and operated at 10 kV.

For transmission electron microscopy, samples were essentially treated as described by Soerensen et al. (2015), with the modification that 2% (v/v) glutaraldehyde was used for fixation and 1% (w/v) uranyl acetate for negative staining. Electron micrographs were taken using a Tecnai 10 transmission electron microscope (FEI Company, Eindhoven, Netherlands) at an accelerating voltage of 80 kV. Digital micrographs were taken with a Megaview G2 CCD camera (Olympus SIS, Münster, Germany).

### Genus-Specific Primer Design and Testing by Conventional PCR

Genus-specific primers were designed for *Eggerthella* and *Slackia* genera based on a clustal W alignment of the 16S rRNA gene of the type species shown in **Table 1**. Clustal W alignment was performed on DNA sequences using the Megalign module [version 10.1.1(3)] of Lasergene (DNASTAR, Inc., Madison, WI, USA). The primers were selected to display maximum sequence difference in 16S rRNA gene nucleotide sequence between the genera in question and the other genera analyzed as controls (**Table 1**). The selected primers were then cross-checked *in silico* as to their specificity by entering them in the ribosomal database project’s (RDP)^1^ Seqmatch tool. The settings used to check for the primers were >1200 bp and KNN factor 20, quality good). Thus, the primers coriofw (5′-GAC GGT ACC TGC AGA AGA AG-3′) and Slackiarev (5′-CCC CGG CTT CGA CGG TGC CGC TT-3′) and Eggfw (5′-TAC TCC TCG CCC CCC TCC TGG-3′) and Eggrev (5′-CTT CTT CTG CAG GTA CCG TC-3′) were used to specifically amplify species of the genera *Slackia* and *Eggerthella* in conventional PCR experiments, in order to check the specificity of the primers, respectively. The Eggfw primer was located in the V2 region of the 16S rRNA gene from 168 to 188 nt relative to the 16S gene of the *E. lenta* DSM 2243 type strain, while the reverse primer (Eggrev) was located between at 447 and 468 (Supplementary Figure S1). The coriofw primer was located between the V3 and V4 region from 456 to 475 nt relative to the sequence in the *Slackia piriformis* YIT 12062 genome. The reverse primer (Slackiarev) was found in the V4 region from 570 to 592 nt (Supplementary Figure S1).

DNA for conventional PCR was isolated according the method of Pitcher et al. (1989). The DNA concentration was measured by spectrophotometry and adjusted to 10 ng/μl. For each PCR reaction, 100 ng of DNA template, 200 nM of the respective dNTPs, 300 nM of the respective primer, 1x Genaxxon PCR buffer (Genaxxon, Ulm, Germany) containing 6 mM MgCl_2_ and 1.5 U of Genaxxon *Taq* polymerase were used. PCR was done in a 50 μl volume with an initial degradation step of 94°C for 5 min followed by 32 cycles of 94°C for 30 s denaturing, 62°C for 30 s annealing (for both *Slackia* and *Eggerthella* PCR) and 72°C for 30 s elongation. PCR products were subjected to electrophoresis on a 1.8% agarose gel for 45 min. The gels were stained with Gelred and the specificity of the reaction (presence of a 140 bp band) was confirmed under UV light using a Fluorchem 5500 (Alpha Innotech, USA).

### Quantitative PCR of *Slackia* and *Eggerthella* Species

In order to generate standard curves for quantitative PCR (qPCR) for specific enumeration of *Slackia* and *Eggerthella* genera in human feces, DNA was isolated from the representative species *S. equolifaciens* DSM 24851^T^ and *E. lenta* strains DSM 15644 and DSM 2243^T^. Before DNA isolation, these bacteria were cultured separately in Wilkins–Chalgren medium (Oxoid, Unipath, Wesel, Germany) containing 0,3 g/L L-cysteine hydrochloride monohydrate (Sigma, Taufkirchen, Germany) and 1 mg/L resazurin (Sigma) at 37°C under anaerobic conditions in Hungate tubes at 37°C for 48 h. After 48 h, DNA was isolated from 2 ml of the culture (>1 × 10^7^ CFU/ml), while 100 μl were used to prepare a tenfold dilution series in the anaerobic chamber for determining the viable count by plate counting on Wilkins–Chalgren agar with L-cysteine hydrochloride monohydrate and resazurin as above. Cell counts were done in triplicate for each culture and were meaned for relating to qPCR results.

DNA from feces was isolated using the Qiagen mini stool kit as described in a different study (Bode et al., 2013). As our study was not clinical in nature, fecal samples were provided voluntarily by colleagues. The DNA was diluted tenfold and was used as template for qPCR, with the bacterial group specific primers. The standard curves were generated from triplicate cultures of each bacterium, as well as triplicate DNA extractions, each from one of the different cultures. Triplicate *C*q-values obtained after qPCR of the standard curve were meaned to obtain a single standard curve. The PCR reaction efficiency (E = 10^1/-^*^S^*– 1, where E = efficiency and S = slope) was calculated from the log-linear part of the standard curve (Töwe et al., 2010).

The qPCR reactions were performed in 96-well plates (Bio-Rad) using a CFX96 quantitative PCR machine (Bio-Rad). The 15 μl reaction mix contained 7.5 μl of iTaq universal SYBR green supermix (Bio-Rad), 3 μl of template DNA, and 200 nM of primers. The annealing temperatures for the qPCR differed for the different genera that were amplified, as this depended on the primers. qPCR amplification conditions were based on a ‘two-step’ qPCR that included one cycle at 95°C for 3 min for initial denaturation, followed by 40 cycles of denaturing at 95°C for 10 s, annealing and extension at the genus-specific temperature at 62°C for 1 min. Melting curve analyses were done immediately at the end of the final amplification cycle to determine the specificity of the PCR reaction, by denaturing from 65 to 94°C while recording the fluorescence. All qPCRs were repeated three times.

### Culturing of Caco-2 Cells in a Transwell System

Adhesion of *S. equolifaciens* DSM 24851^T^ and *E. lenta* strains DSM 15644 to Caco-2 cells were tested in cell culture. For this, Caco-2 cells were seeded at 0.4 × 10^6^ cells onto 9 of 12 transwell filter supports (Costar, Corning, NY, USA), respectively, and grown for 3 weeks with growth medium changes every 2–3 days. Cells were cultured at 37°C under 5% CO_2_ in Essential Eagle’s Medium (Invitrogen, Karlsruhe, Germany), supplemented with 10% (v/v) heat-inactivated (56°C, 30 min) fetal calf serum (FCS, PAA Laboratories GmbH, Cölbe, Germany), 100 U/ml penicillin G (Invitrogen, Karlsruhe, Germany) and 100 μg/ml streptomycin sulfate (Invitrogen, Karlsruhe, Germany).

Before the adhesion experiments, the Caco-2 cell monolayer was tested for integrity by fluorescein rejection. For this, fluorescein sodium salt (Fluka, Steinheim, Germany) stock solution (2.5 mg/100 ml PBS with Ca and Mg, pH 7.4; Invitrogen, Karlsruhe, Germany) was diluted 1:10 (v/v) with PBS buffer (pH 7.4; Invitrogen). This solution was added to each well of the transwell insert (apical compartment) to measure the transport across the monolayer or the rejection of fluorescein. Three of 12 wells which did not contain cells served as a negative control to measure the free diffusion of fluorescein to the basolateral side of the transwell. Plates were incubated for 40 min at 37°C in the cell culture incubator with 5% CO_2._ Then, the concentration of fluorescein was estimated in both the apical and basolateral chambers using a microplate reader (Pherastar BMG Labtech, Ortenberg, Germany); excitation 485, emission 520 nm. All fluorescence measurements were done in triplicate and only inserts with low fluorescein permeability were used for adherence assay, as this indicated that cells were confluent. After determining the integrity of the monolayer, cells were washed twice with 2 ml PBS buffer and then incubated for a further 2 days with cell culture medium without antibiotics.

### Adhesion Assays

All equipment, solutions and plates used for cultivating anaerobic bacteria were pre-incubated in an anaerobic chamber one day prior to the adhesion experiment. Four aliquots of each 2 ml (v/v) fresh 48 h culture, of either the *Slackia* or the *Eggerthella* strain, were each washed twice by centrifugation at 7 500 × g for 5 min. The pellets were resuspended in 1.5 ml PBS-Ca/Mg. Three of the four 1.5 ml aliquots of bacterial suspension thus prepared were each used to inoculate three wells in a 12 transwell plate used in the adhesion assay, thus serving as a triplicate sample. In addition, the last bacterial suspension was diluted 1:10 in PBS-Ca/Mg in another four aliquots to inoculate an additional three wells (another triplicate sample) and to prepare a further 1:100 dilution in three aliquots which were then used to inoculate a further three wells. Three wells of the 12-well plate were used as control with PBS-Ca/Mg which did not contain bacteria. The plate was incubated for 4 h under anaerobic conditions (80% N_2_, 10% CO_2_, and 10% H_2_). After 4 h incubation, the plate was washed with PBS-Ca/Mg twice in both compartments (upper cups and bottom wells) and the Caco-2 cell monolayer in the upper cups, which potentially had bacteria bound to the surface, were detached from the cup membrane with 300 μl of a 0.1% (w/v) trypsin solution for 5 min in 37°C under anaerobic conditions. These wells were washed two further times by pipetting with 600 μl PBS-Ca/Mg followed by centrifugation at 7,500 × g for 5 min, in order to harvest the Caco-2 cells and bacteria. The pellet was then resuspended in 1ml PBS Ca/Mg, serially diluted in PBS without Ca/Mg using a tenfold dilution which was followed by plating onto Wilkins–Chalgren agar with L-cysteine hydrochloride monohydrate and resazurin to enumerate bacteria. These agar plates were incubated in an anaerobic chamber at 37°C under anaerobic condition (described above) for 48 h.

## Results

### Morphological Characterization by Electron Microscopy

**FIGURE 1:**
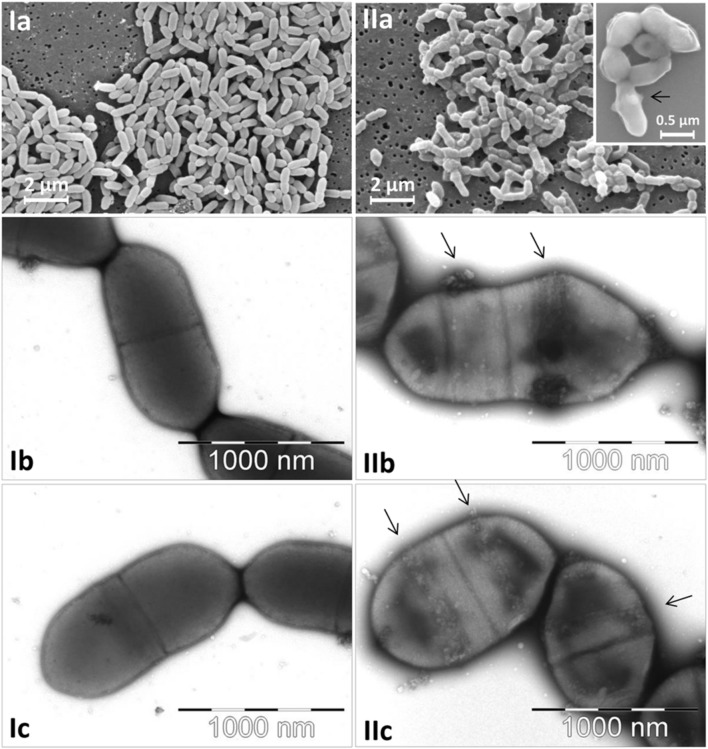
**Micrographs of *Eggerthella lenta* DSMZ 15644 cells (Ia–c) and of *Slackia equolifaciens* DSMZ 24851^T^ cells (IIa–c) captured by scanning electron microscopy at low and high magnification (Ia and IIa with insert) and by transmission electron microscopy at high magnification (Ib,c; IIb,c).** The arrows indicate characteristic ribbon bands of extracellular matrix material on the surface of *S. equolifaciens* cells.

The morphological characteristics of *E. lenta* DSMZ 15644 and *S. equolifaciens* DSMZ 24851^T^ are shown in **Figure 1**. Cells of both cultures were small and were growing in short chains. Both cultures did not reveal flagella. The *E. lenta* culture was composed of uniformly growing short and thin rods, while *S. equolifaciens* cells exhibited a remarkably pleomorphic phenotype (i.e., irregularly shaped cells of different sizes). As seen at high magnification on transmission and scanning electron micrographs, *S. equolifaciens* cells revealed characteristically shaped “ribbons” of extracellular material (see arrows in **Figure 1**, IIb,c), which are probably synthesized at new cell division regions on the cell wall. No extracellular material was detected on the *E. lenta* cells.

### Genus-Specific, Conventional PCR for Selective Amplification of *Eggerthella* spp. and *Slackia* spp.

**FIGURE 2:**
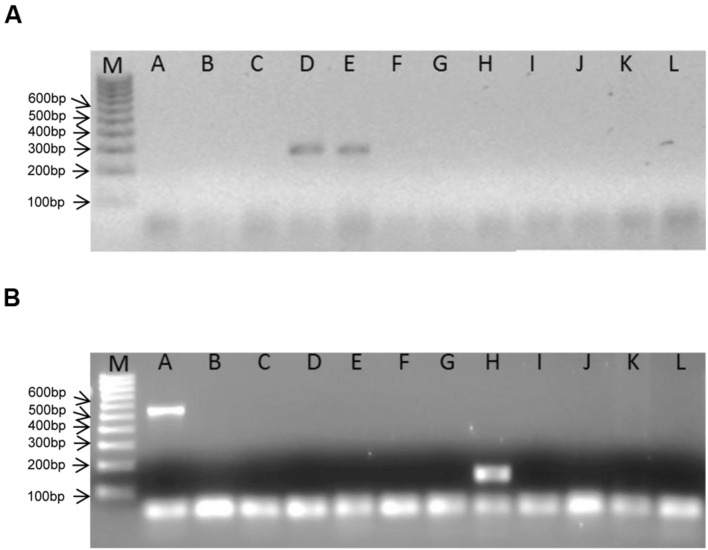
**Bands obtained in genus-specific, conventional PCR with *Eggerthella* (A)- and *Slackia* (B)- specific primers and DNA from different coriobacterial species.** M: 100 bp ladder DNA size marker. A: *Atopobium parvulum* DSM 20469^T^, B: *Adlercreutzia equolifaciens* DSM 19450^T^, C: *Collinsella intestinalis* DSM 13280^T^, D: *Eggerthella lenta* DSM 2243^T^, E: *Eggerthella lenta* DSM 15644, F: *Enterorhabdus mucosicola* DSM 19490^T^, G: *Gordonibacter pamelaeae* DSM 19378^T^, H: *Slackia equolifaciens* DSM 24851^T^, I: *Escherichia coli* DH5alpha, J: *Faecalibacterium prausnitzii* DSM 17677, K: *Ruminococcus obeum* DSM 25238^T^, L: *Lactobacillus plantarum* BFE 5092.

The genus-specific PCR primers for the specific detection of *Eggerthella* species yielded PCR amplicons of the expected size of 325 bp and is shown for the two *Eggerthella* reference strains used in this study (**Figure 2A**). The optimal condition for the *Eggerthella* genus-specific reaction was at a primer annealing temperature of 60°C for 30 s. A PCR product of an expected size of 140 bp was also obtained with the *Slackia* genus-specific primers when using DNA from *S. equolifaciens* (**Figure 2B**). The PCR reaction was also done at a primer annealing temperature of 60°C. Both genus-specific primers targeted the 16S rRNA gene and were quite specific, as DNA from species of other *Actinobacteria* genera (e.g., *Adlercreutzia, Collinsella*, and *Gordonibacter*) or other gut associated bacteria (*Bacteroides, Ruminococcus, Escherichia, Lactobacillus*), when used as a matrix, did not yield a PCR product (**Figures 2A,B**). Only in the case of DNA from *Atopobium parvulum* DSM 20469 was a, non-specific PCR product of 550 bp, i.e., more than the expected size, detected. The difference in amplicon size between *Slackia* and *Atopobium* strains could be easily observed in both normal gel electrophoresis or by a low additional peak in melting curve analysis in qPCR (see below). Thus, the PCR condition and the primers tested for the genus-specific detection and enumeration of *Slackia* and *Eggerthella* were deemed acceptable for further qPCR analysis to enumerate these bacteria in feces samples.

### Enumeration of *Slackia* and *Eggerthella* Species with qPCR

**FIGURE 3:**
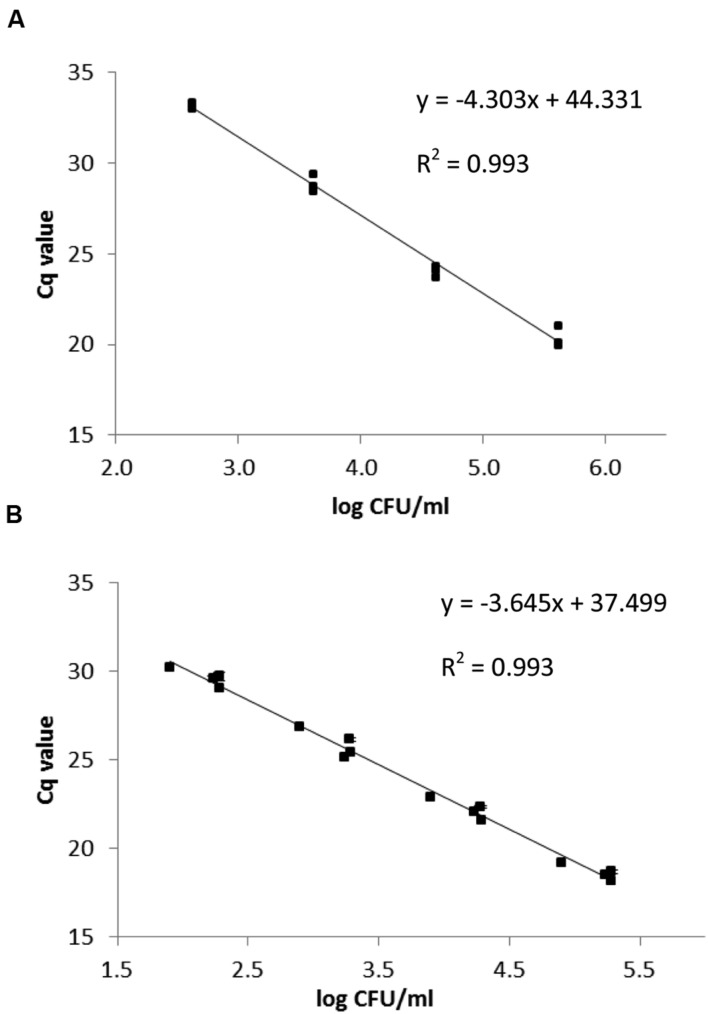
**qPCR standard curves relating the *C*q-value to log CFU/ml for *E. lenta* strains DSM 2243^T^ and DSM 15644 (A) and *S. equolifaciens* DSM 24851^T^ (B).** The data are based on triplicate independent assessments.

*Eggerthella lenta* DSM 2243^T^, *E. lenta* DSM 15644 and *S. equolifaciens* DSM 24851^T^ were grown in pure culture in medium 339 at 37°C under anaerobic conditions in order to produce standard curves that relate bacterial counts with *C*q-values obtained in the qPCR. The correlations between Cq values and the CFU/ml of *Slackia* and *Eggerthella* cell number in standard curves were linear (**Figure 3**). The standard curve obtained for *Eggerthella* spp. showed that the minimum number of *Eggerthella* spp. that could be detected was ca. 4.0 × 10^2^ CFU/ml (**Figure 3A**) and the qPCR efficiency was 70.8%. This PCR efficiency was lower, and may have been a result of the fact that 2 different *Eggerthella* spp. were used for generating a common standard curve. Alternatively, primer design or salt concentrations may have caused this and could possibly be improved in further studies. The minimum number of *Slackia* bacteria which could be detected by qPCR was ca. 1 × 10^2^ CFU/ml and the qPCR amplification efficiency for the *Slackia* spp. was 88.1% (**Figure 3B**).

**FIGURE 4:**
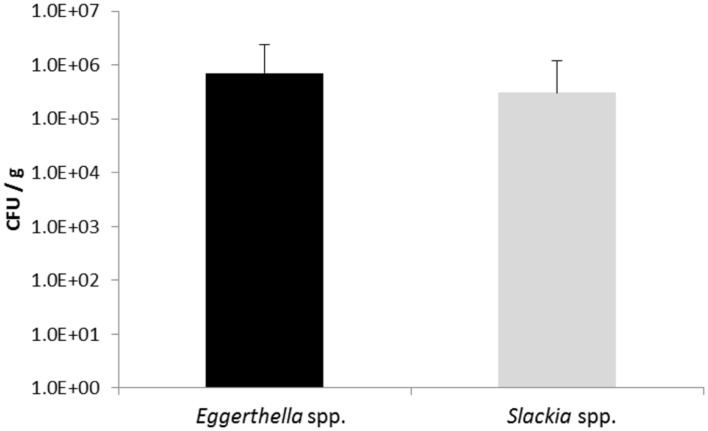
**Bacterial counts calculated from triplicate qPCR of all 19 individual human feces samples.** The standard deviation is shown. Black bar: mean values of *Eggerthella* spp., gray bar: mean values of *Slackia* spp.

In order to determine the number of *Slackia* and *Eggerthella* species in feces samples, the human feces DNA from nineteen volunteers obtained in two previous studies (Barth et al., 2012; Bode et al., 2013) was isolated using a DNA mini stool kit (Qiagen) and used as template in qPCR with iTaq SYBRGreen (Bio-Rad). Each bacterial count was done in triplicate and the standard error was determined. The number of bacteria detected with the *Eggerthella* genus- specific primers ranged from 1.9 × 10^3^ and 7.0 × 10^6^ CFU/g feces and the numbers of *Slackia* bacteria detected with the *Slackia*-specific primers was observed to range between 1.8 × 10^3^ to 4.0 × 10^6^ CFU/g feces, depending on individuals (**Table 2**). The average *Eggerthella* and *Slackia* counts observed for the 19 individuals were 7 × 10^5^ and 3.1 × 10^5^ CFU/g, respectively, as determined by qPCR (**Figure 4**). From **Table 2** it was noted that 14 of 19 individuals (74%) and 15/19 (79%) individuals had counts higher than 1 × 10^4^ CFU/g *Eggerthella* and *Slackia* spp., respectively. Thus, approximately three-quarters of tested individuals had *Eggerthella* and *Slackia* counts of more than 1 × 10^4^ CFU/g in their feces, while approx. half of tested individuals had counts of more than 1 × 10^5^ CFU/g in the feces.

**Table 2:** Mean number of *Eggerthella* and *Slackia* spp. in fecal samples (CFU per gramwet feces) of 19 volunteers.

Sample from volunteer	Mean no. of *Eggerthella* spp. (CFU/g feces)	Standard error	Mean of *Slackia* spp. (CFU/g feces)	Standard error
01	2.0 *E* + 03	3.7 *E* + 02	1.0 *E* + 05	1.4 *E* + 03
02	5.5 *E* + 04	5.2 *E* + 03	7.1 *E* + 05	1.6 *E* + 04
03	7.0 *E* + 05	6.8 *E* + 04	3.3 *E* + 04	2.7 *E* + 03
04	1.5 *E* + 04	3.6 *E* + 03	6.7 *E* + 03	2.9 *E* + 03
05	3.1 *E* + 05	5.2 *E* + 04	1.8 *E* + 03	4.8 *E* + 02
06	5.6 *E* + 04	2.5 *E* + 03	2.5 *E* + 05	3.3 *E* + 04
07	1.9 *E* + 03	1.1 *E* + 03	1.3 *E* + 04	1.2 *E* + 03
08	9.7 *E* + 03	8.9 *E* + 02	1.2 *E* + 05	1.5 *E* + 04
09	3.8 *E* + 03	5.5 *E* + 02	2.3 *E* + 03	1.5 *E* + 03
10	2.5 *E* + 04	1.8 *E* + 03	4.0 *E* + 06	4.9 *E* + 05
11	7.0 *E* + 06	2.1 *E* + 05	1.4 *E* + 05	9.8 *E* + 03
12	4.2 *E* + 05	6.2 *E* + 03	3.4 *E* + 04	3.3 *E* + 03
13	7.1 *E* + 05	4.8 *E* + 03	2.1 *E* + 05	8.4 *E* + 03
14	3.9 *E* + 06	2.2 *E* + 05	4.0 *E* + 04	3.2 *E* + 03
15	6.6 *E* + 03	3.9 *E* + 03	4.6 *E* + 04	2.5 *E* + 03
16	1.6 *E* + 04	2.3 *E* + 03	2.4 *E* + 04	4.9 *E* + 03
17	8.2 *E* + 04	5.1 *E* + 03	1.1 *E* + 05	9.8 *E* + 04
18	5.0 *E* + 05	3.9 *E* + 04	2.0 *E* + 04	1.2 *E* + 03
19	2.1 *E* + 04	3.4 *E* + 03	8.9 *E* + 03	1.2 *E* + 03

*The standard error of triplicate counts is indicated*.

### Adhesion to Caco-2 Cells

**FIGURE 5:**
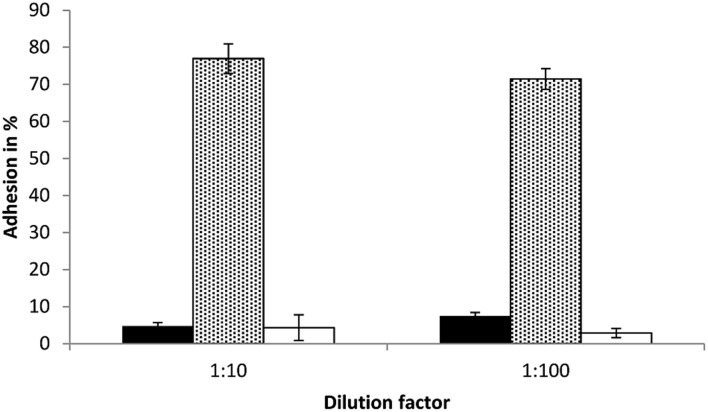
**Bacterial adhesion (%) to Caco-2 cells in transwell plates as determined in triplicate independent analyses.** Black bar: *S. equolifaciens* DSM 24851^T^, dotted gray bar: *E. lenta* DSM 15644, white bar: *Lactobacillus plantarum* 299v (control probiotic strain). The error bar indicates the standard deviation.

In this study, the adhesion ability of *S. equolifaciens* DSM 24851^T^ and *E. lenta* DSM 15644, two strict anaerobic bacteria, to intestinal Caco-2 cells in a transwell plate under anaerobic conditions, was determined. *E. lenta* DSM 15644 showed a high adhesion capacity to Caco-2 cells at a level of 74.2% adherence (76.9% in test with bacterial cells diluted 1:10 and 71.5% in test with bacterial cells diluted 1:100; **Figure 5**). *S. equolifaciens* DSM 24851^T^ on the other hand showed only low adhesion capacity for adherence to these cells as only 4.7% (1:10 dilution) and 7.5% (1:100 dilution) of bacterial cells remained bound and could be recovered after washing of Caco-2 cells. The probiotic *L. plantarum* strain 299v was used as a control, as it is a well-known probiotic strain previously reported to adhere relatively well (when compared to other probiotic lactic acid bacteria) to Caco-2 cells (Jacobsen et al., 1999) in adhesion assays that were not done in transwell plates. The adhesion capacity of this strain in our study ranged from 2.9 to 4.3% for the duplicate assessments with either 1:10 or 1:100 diluted bacterial inocula. The differences in percentages between the tests with bacteria from different dilutions are probably the result of deviation in counts between replicate assessments.

## Discussion

Coriobacteria are common members of the human and animal gut microbiota (Eckburg et al., 2005; Arumugam et al., 2011) and amongst these, *Collinsella* and *Eggerthella* are among the top five most abundant genera of the phylum *Actinobacteria* in the human gut. The genus *Collinsella* by itself even featured among the top six most abundant genera, being present in 32 out of 35 gut microbiota samples investigated at a sequence abundance of 1.8% of total microbiota (Arumugam et al., 2011). Louis et al. (2007) reported that bacteria of the *Atopobium* cluster, belonging to the *Atopobiaceae* of the order *Coriobacteriales*, occur at an abundance of 2.1 to 11.9% of total bacteria of colonic bacteria. The *Actinobacteria*, however, have in numerous studies been described to be underrepresented or to remain undetected in clone libraries of the human gut microbiota (Wilson and Blitchington, 1996; Suau et al., 1999; Harmsen et al., 2000; Koenig et al., 2011; Maukonen et al., 2012; Sim et al., 2012; Thorasin et al., 2015). Possible explanations for this may be the relative hydrophobicity of the cell walls and high mol% G+C content in the DNA, which leads to difficulties with DNA isolation and PCR amplification of clone libraries due to bias in PCR primers (Thorasin et al., 2015).

This study aimed to develop a quantitative PCR assay specific for the genera *Slackia* and *Eggerthella*. For this, primers were developed based on an *in silico* approach and tested for their relative specificities. The primers and amplification conditions developed proved quite specific for specific amplification of the genera *Slackia* and *Eggerthella*, for which so far, no specific PCR amplification primers have been developed. When the same primers were used for quantitative PCR, a standard curve was first established with pure cultures of *S. equolifaciens* and *E. lenta* for which PCR signals were correlated with viable cell counts. A previous qPCR study based on enumeration of bacteria from pure cultures showed that the qPCR efficiencies of the reactions ranged from 80 to 87% in different dilutions of the matrix DNA (Töwe et al., 2010). These amplification efficiencies were considered suitable for deriving bacterial cell counts from qPCR standard curve data (Töwe et al., 2010); therefore, the similar amplification efficiencies determined in this study, also indicated the suitability of the standard curves based on the 16S rRNA gene primers for enumeration of *Slackia* and E*ggerthella* species by qPCR for determining genus-specific bacterial counts in this study.

Previous studies on numbers of coriobacteria in human feces which were based on FISH using a ‘*Atopobium* cluster’-specific probe, which targets the genera *Collinsella, Coriobacterium, Atopobium*, and *Cryptobacterium*, showed that these bacteria occur in the feces of humans at levels above ca. 5 × 10^9^ up to 1.1 × 10^10^ per gram dry weight (Harmsen et al., 2000; Thorasin et al., 2015). However, these studies did not provide any information on the specific numbers of *Slackia* and *Eggerthella* bacteria present in the feces, as the probes used in the FISH experiments did not hybridize to bacteria of these genera. As these bacteria, however, may play an important role in the metabolism of bioactive compounds such as daidzein and resveratrol (Matthies et al., 2009; Bode et al., 2013), this study aimed to determine their numbers in human feces. Our results show that the numbers of *Slackia* and *Eggerthella* were considerably lower than the reported numbers of the ‘*Atopobium*’– or *Collinsella*-groups (Harmsen et al., 2000; Thorasin et al., 2015), at an average of about 105 to106 CFU/g feces. Tsuji et al. (2010) developed a *Slackia* primer based on the 16S rRNA sequence of the *Slackia* sp. strain NATTS, which not only covered strain NATTS, but also related strains. When this primer was used, they were able to enumerate this *Slackia* sp. at a population level of log 6.4 cells per gram wet feces (Tsuji et al., 2010). This is similar to the numbers found in this study. For some individuals, the numbers of these bacteria determined in our study were much lower at levels of about 10^3^ CFU/g. However, in the case of our *Slackia*-specific primers, the RDP Sequence match showed that these were specific only for *S. equolifaciens, S. piriformis* and *S. faecicanis* and thus did not cover the complete *Slackia* genus. Thus these primers could have missed *S. isoflavoni*-convertans, which was enumerated in the study by Tsuji et al. (2010). The use of the primers in this study may therefore possibly underreport the numbers of all *Slackia* spp. in human feces. It should be considered, however, that the extent of underreporting would be difficult to judge, especially as the *Slackia* reverse primer, which is responsible for the specificity of the amplification, differed in only two nucleotides out of the 23 nt primer. This primer in conventional PCR did yield a specific product. On the other hand, the primers also amplified *Atopobium parvulum*, and where thus not strictly genus specific either. Clearly, this should be more closely investigated with more strains, also related, non-*Slackia* strains, in a further study.

Schwiertz et al. (2000) used a rRNA oligonucleotide probe to determine the numbers of *E. lenta* in human feces and found these bacteria to be present at a range of log 8.1–8.5. This is clearly higher than the counts determined in this study, which ranged from log 3 to log 6. Reason for this may be that the probe used by Schwiertz et al. (2000) detected also other *Eggerthella* species, which at the time had not been described or investigated, as in the report of Schwiertz et al. (2000) the *Eggerthella lenta* was taxonomically still considered as *Eubacterium lentum*. The Eggfw/Eggrev primers used in this study according to the RDP Sequence match targeted *Eggerthella lenta* and *E. sinensis*. Thus the primer pair used in our study may also underreport the amounts of *Eggerthella* in human feces samples. As for the case of the *Slackia* primers, the specificity should also be investigated with more *Eggerthella* species and other related, non-*Eggerthella* species, in more detailed further studies. Furthermore, the *Eggerthella* PCR products in the conventional PCR showed weaker bands when compared to the band obtained with the *Slackia* primers, therefore the PCR conditions could still be optimized further for the *Eggerthella* PCR.

Thorasin et al. (2015) showed that the human fecal ‘*Atopobium-cluster*’ population, especially species of the genera *Collinsella*, *Gordonibacter*, *Olsenella*, and *Paraeggerthella*, was quite stable over a three months period. However, these data did not reveal whether species of the genera *Slackia* and/or *Eggerthella* would show a similar stable composition. As these occur in lower numbers, this might hypothetically not be the case. Furthermore, the numbers of *Slackia* and *Eggerthella* in our study seem to vary considerable from between 10^3^ to 10^6^ CFU/g in different individuals in this study, indicating that the health-associated functions of these bacteria, e.g., in converting daidzein to equol, may be functioning at different levels of effectiveness in different individuals that contain these bacteria in their microbiota. Given the relative importance of these bacteria in gastrointestinal health, this clearly would warrant further investigation.

The adhesion assays with Caco-2 cells in this study showed that cells in the transwell plates grew well to reach confluence within 21 days and that 1:10 or 1:100 dilutions of the bacterial cultures were suitable for use in binding assays. The novelty of this study was that incubation of Caco-2 cells with bacteria was done under strictly anaerobic conditions and using differentiated polarized Caco-2 cells on permeable membranes. These conditions are similar to an *in vivo* situation: Bacteria were applied to the apical side of Caco-2 cells, but the basolateral side of the cells was incubated with the bacteria-free basolateral compartment medium. Furthermore, the integrity and differentiation of Caco-2 cells were determined by fluorescein (a compound known to have low permeability across the integral Caco-2 cell monolayer) and measurement of transepithelial electrical resistance (Masungi et al., 2004), as well as electronic microscopy (formation of microvilli; SEM as done in this study with similar method). Our pilot experiments done with Caco-2 cells incubated under anaerobic conditions without bacteria for 4 h resulted in only a slight decrease in viability of Caco-2 cells as tested by trypan blue exclusion (results not shown). This is the reason for incubating the bacteria on cells during the adhesion studies for 4 h. The results showed that *L. plantarum* 299v showed low binding to Caco-2 cells in comparison with *Eggerthella* bacteria (**Figure 4**). Although previously described to bind well, the adhesion behavior in this study may be different because of differences in methodology to determine binding between the two studies. Jacobsen et al. (1999) counted bacteria in 20 microscopic fields, instead of determining the percentage of bacteria adhering by viable plate counting, as was done in this study. In addition, the ratio of bacteria/Caco-2 cells used may have been different to that used in this study.

*Eggerthella lenta* showed high adhesion of up to 80% to gut epithelial cells in cell culture in this study, while the binding ability of *S. equolifaciens* was low, as only less than 10% of cells bound to Caco-2 cells. We recently sequenced the genome of *S. equolifaciens* DSM 24851^T^ at a commercial sequencing facility that used an Illumina Miseq for sequencing. The sequence data were aligned into contigs and scaffolds and sent for annotation using the RAST (Rapid Annotation using Subsystem Technology) server (Aziz et al., 2008). There were still numerous gaps in the chromosomal sequence, and more bioinformatic analyses need to be performed, but preliminary results show that *S. equolifaciens* DSM 24851^T^ does not possess genes for fimbriae or pili, which was also evident from our electron microscopy results. Interestingly, on the cells extracellular material arranged in “ribbons” could be seen, especially in areas where the cell was dividing, suggesting the presence of extracellular matrix proteins coating the cells (**Figure 1**, IIa insert). Indeed, in the genome sequence of *S. equolifaciens*, genes for extracellular matrix components were present.

This study is the first to report on the approximate numbers of coriobacteria belonging to the genera *Slackia* and *Eggerthella*, especially the species *S. equolifaciens* and *E. lenta* for which the primers were more specific. These are bacteria which may play an important role in the gut health of human beings. Our results also suggest a distribution of these species to specific compartments, i.e., the gut lumen in the case of *Slackia* and the gut wall in the case of *Eggerthella*. However, basing this assumption on adhesion data in cell culture would be speculative, and thus this aspect should be further investigated.

## Author Contributions

G-SC planned and carried out experiments on qPCR, cell culture and adhesion of coriobacteria. DB and HN carried out TEM and REM, respectively. FR carried out adhesion experiments in cell culture with G-SC. ME carried out qPCR with G-SC. MH cultivated bacteria anaerobically. KB developed the anaerobic cell culture with G-SC and tested cell survival. CF designed and was responsible for research with G-SC and also designed custom primers for qPCR with G-SC and ME.

## Conflict of Interest Statement

The authors declare that the research was conducted in the absence of any commercial or financial relationships that could be construed as a potential conflict of interest.

## Footnotes

1 https://rdp.cme.msu.edu/
