# Supplementary material for: Quantification of Slackia and Eggerthella spp. in Human Feces and Adhesion of Representatives Strains to Caco-2 Cells
Source: Front Microbiol. 2016 May 9;7:658. doi: 10.3389/fmicb.2016.00658 (PMC4860493; doi:10.3389/fmicb.2016.00658)
Supplement: Supplementary file 1 [file Presentation_1.PPTX]

## Slide 1
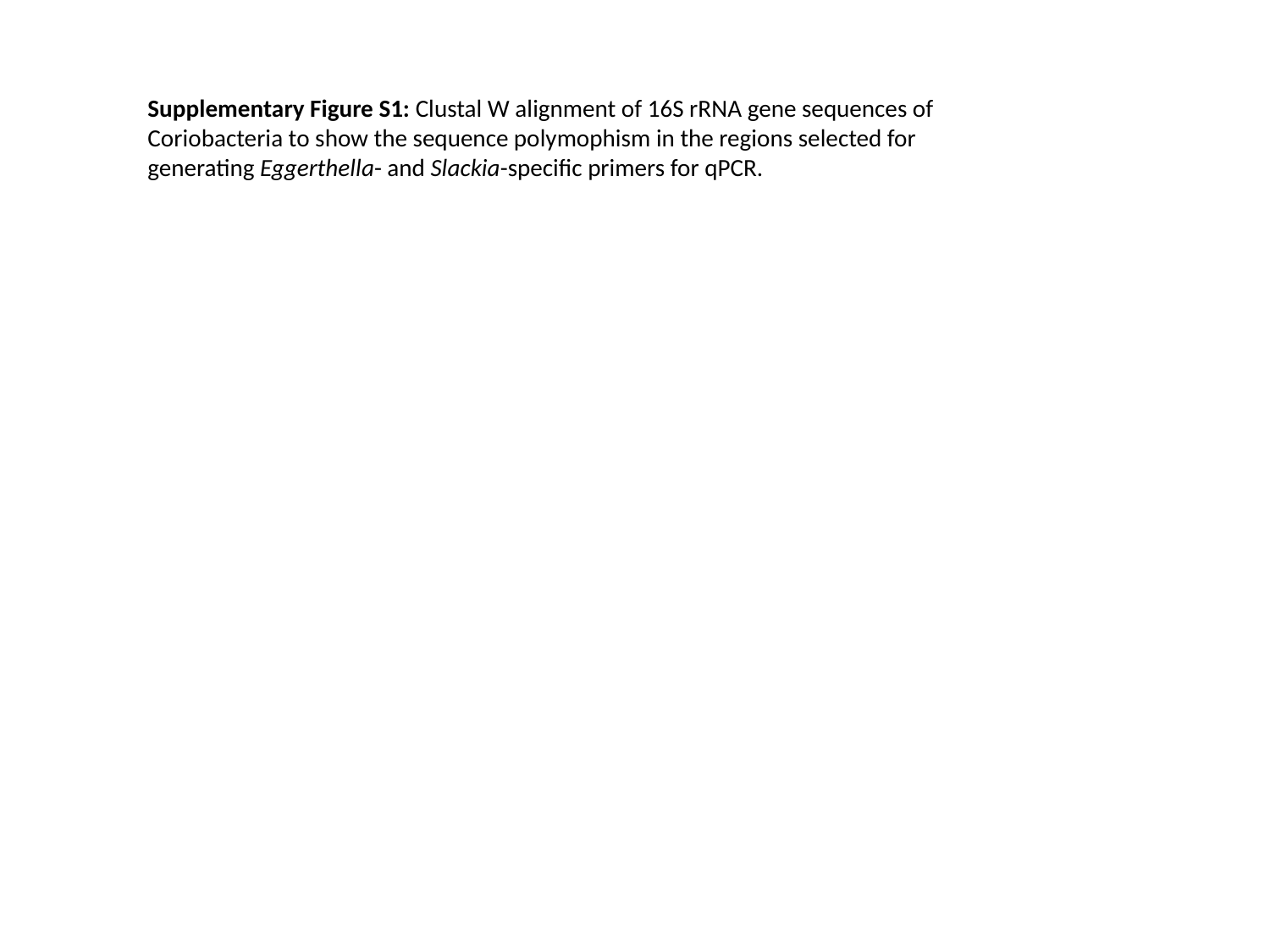

Supplementary Figure S1: Clustal W alignment of 16S rRNA gene sequences of Coriobacteria to show the sequence polymophism in the regions selected for generating Eggerthella- and Slackia-specific primers for qPCR.

## Slide 2
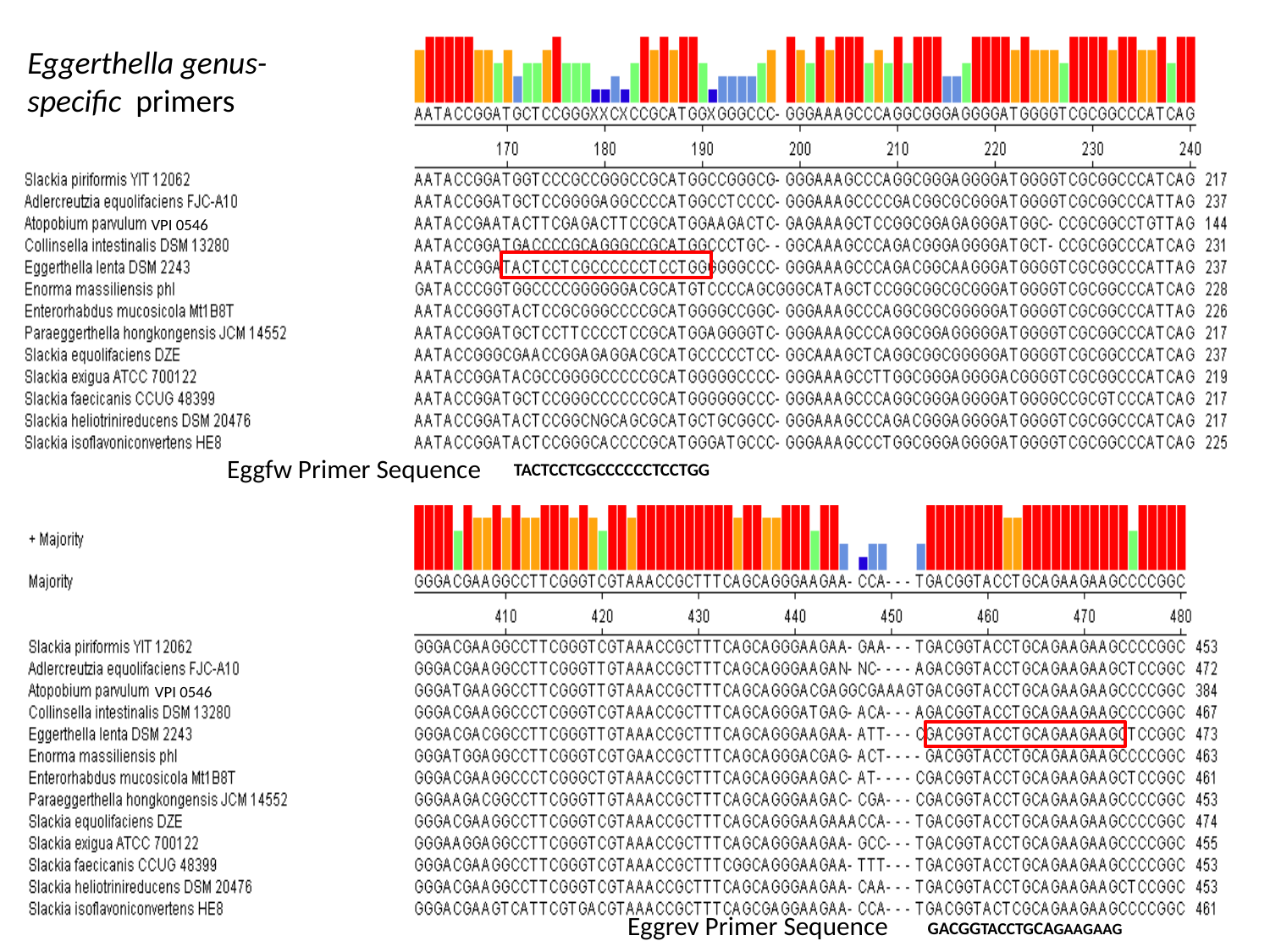

Eggerthella genus-specific primers
VPI 0546
Eggfw Primer Sequence
TACTCCTCGCCCCCCTCCTGG
VPI 0546
Eggrev Primer Sequence
GACGGTACCTGCAGAAGAAG

## Slide 3
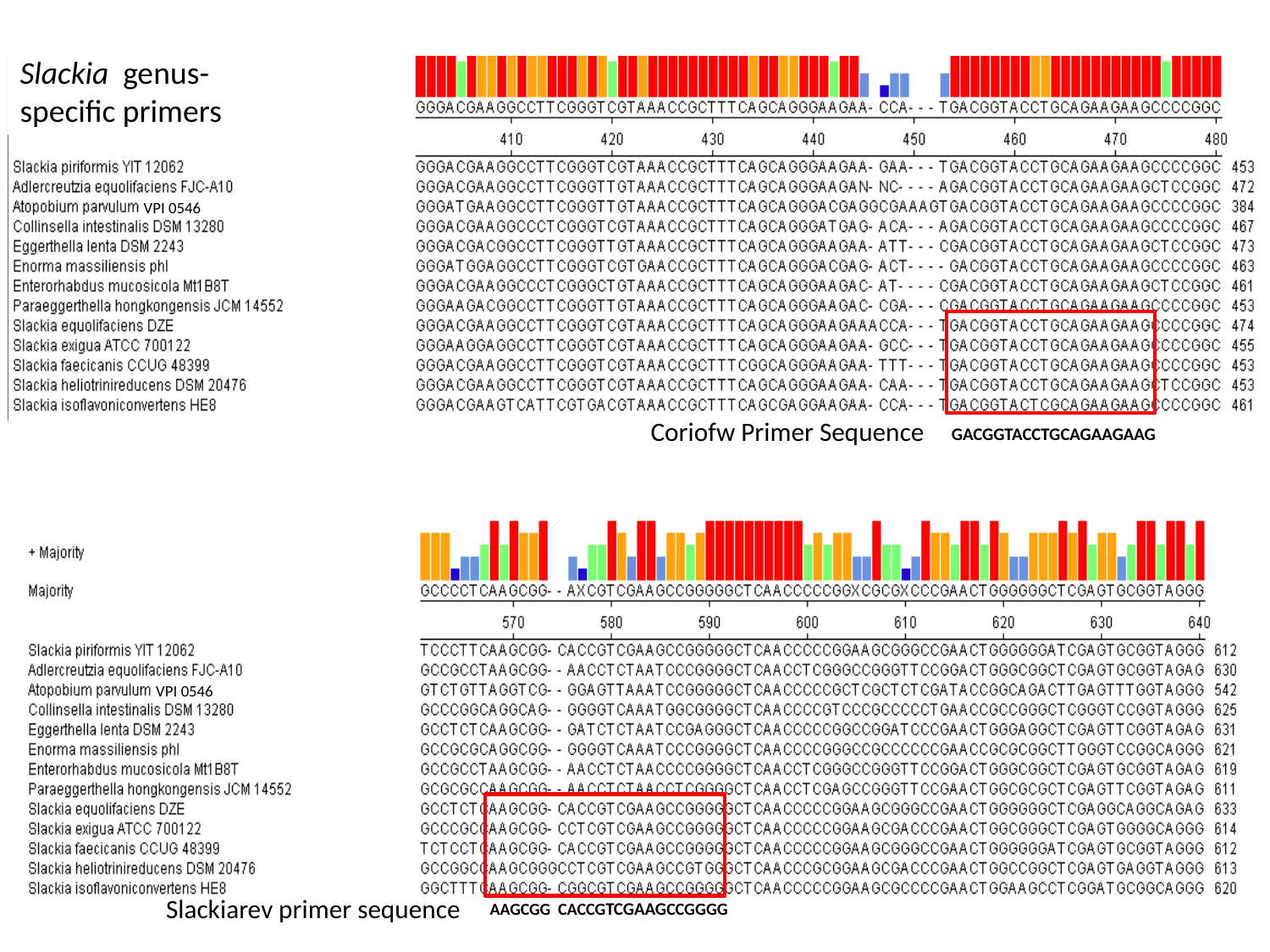

Slackia genus-specific primers
VPI 0546
Coriofw Primer Sequence
GACGGTACCTGCAGAAGAAG
VPI 0546
Slackiarev primer sequence
AAGCGG CACCGTCGAAGCCGGGG
